# Supplementary material for: Drug repurposing for aging research using model organisms
Source: Aging Cell. 2017 Jun 16;16(5):1006–15. doi: 10.1111/acel.12626 (PMC5595691; doi:10.1111/acel.12626)
Supplement: Supplementary file 7 — Data S1 Zip‐Archive of all report cards. [file ACEL-16-1006-s007.zip › RC_TSZ.pdf]

TSZ

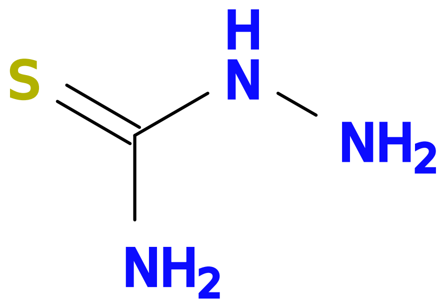

Database identifiers

ChEMBLCompound	CHEMBL256250
ZINC	ZINC08830546
eMolecules	532388

Ranking

	Rank	Score
Drosophila	362/697	0.415
C. elegans	NA	NA

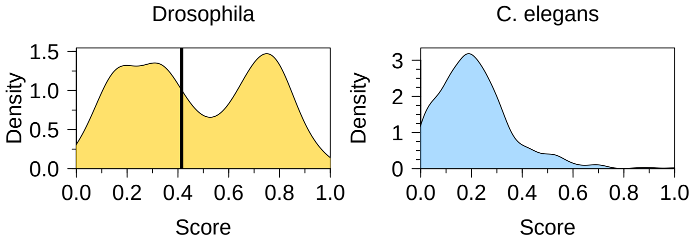

	Ageing implication	Domain conservation	Binding site conservation	Binding affinity	Bioavailability	Lipinski	Promiscuity	Purchasability	Drug approval	**Total**
Drosophila	0.624	0.802	1.0	0.699	(0.9)	0.0	-0.0	0.1	0.0	0.415
C. elegans	NA	NA	NA	NA	NA	NA	NA	NA	NA	NA

Names

· Hydrazinecarbothioamide
· Thiosemicarbazide

Roles

ChEBI entry None has no roles

Status

Approved drug (according to ChEMBL)	No
Number of Rule of 5 violations	0
Binding affinity to original target in log units (RF-Score prediction)	5.84
Burns *C. elegans* bioavailability prediction	-2.98

Compound Target Characteristics

Arginase-1

Best gene implication in ageing for this target family came from gene P07824 via mapping the annotation from RGD 2150 annotated in RGD 2014-03-11. Annotation GO 7568 (aging) was Inferred from Expression Pattern

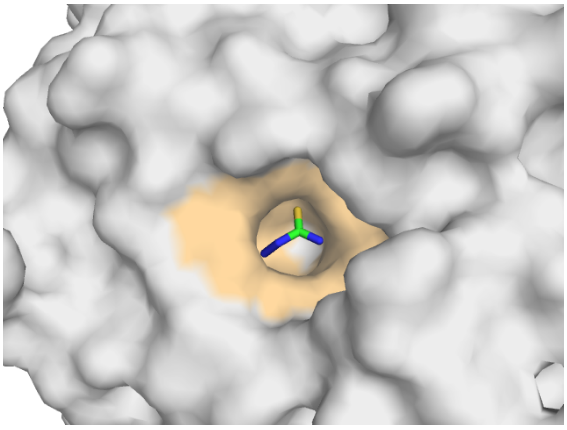

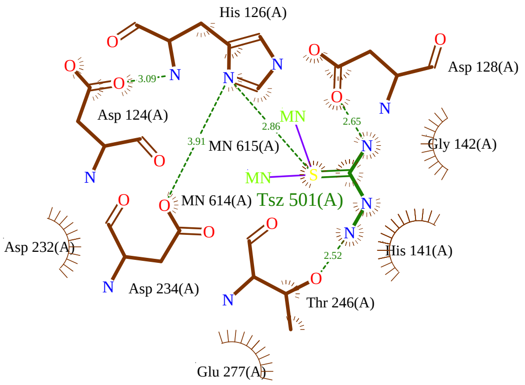

protein	amino acids contacts (binding site)
PDB:2pho:chainA:P05089	D H D H G D D T E
sp:P05089:ARGI1_HUMAN	D H D H G D D T E
sp:P07824:ARGI1_RAT	D H D H G D D T E
sp:Q61176:ARGI1_MOUSE	D H D H G D D T E
tr:Q9NHA5:Q9NHA5_DROME	D H D H G D D T E
tr:O76895:O76895_DROME	D H D H G D D T E
sp:P00812:ARGI_YEAST	D H D H G D D T E

	whole protein		domain-based		contact-based	
protein	ident	simil	ident	simil	ident	simil
PDB:2pho:chainA:P05089	1.0	1.0	1.0	1.0	1.0	1.0
sp:P05089:ARGI1_HUMAN	1.0	1.0	1.0	1.0	1.0	1.0
sp:P07824:ARGI1_RAT	0.87	0.96	0.87	0.96	1.0	1.0
sp:Q61176:ARGI1_MOUSE	0.87	0.96	0.87	0.96	1.0	1.0
tr:Q9NHA5:Q9NHA5_DROME	0.35	0.71	0.36	0.74	1.0	1.0
tr:O76895:O76895_DROME	0.34	0.7	0.36	0.74	1.0	1.0
sp:P00812:ARGI_YEAST	0.39	0.71	0.4	0.72	1.0	1.0

arg (FBgn0023535) associated phenotypes

partially lethal - majority die, some die during pupal stage

(Information from FlyBase)

arg (UniProt:Q9NHA5) annotation

Cofactor: Mn(2+) Evidence=(RuleBase:RU361159); Note=Binds 2 manganese ions per subunit. (RuleBase:RU361159);
Pathway: Nitrogen metabolism; urea cycle; L-ornithine and urea from L-arginine: step 1/1. (RuleBase:RU361159).

(Information from UniProt)

arg (UniProt:O76895) annotation

Cofactor: Mn(2+) Evidence=(RuleBase:RU361159); Note=Binds 2 manganese ions per subunit. (RuleBase:RU361159);
Pathway: Nitrogen metabolism; urea cycle; L-ornithine and urea from L-arginine: step 1/1. (RuleBase:RU361159).

(Information from UniProt)
